# ERGA-BGE reference genome of the Mediterranean monk seal ( *Monachus monachus*), an IUCN Vulnerable species

**DOI:** 10.12688/openreseurope.23936.1

**Published:** 2026-06-09

**Authors:** Tereza Manousaki, Panagiotis Dendrinos, Katerina Vasileiadou, Xenia Sarropoulou, Costas S. Tsigenopoulos, Thanos Dailianis, Eleni Tounta, Nikitas Vogiatzis, Anastasia Komnenou, Astrid Böhne, Rita Monteiro, Rosa Fernández, Nuria Escudero, Ignas Bunikis, Christian Tellgren-Roth, Mai-Britt Mosbech, Guilherme B. Dias, Olga Vinnere Pettersson, Chiara Bortoluzzi

**Affiliations:** 1 Institute of Marine Biology Biotechnology and Aquaculture, Heraklion, Hellenic Centre for Marine Research, Crete, 70014, Greece; 2 MOm/Hellenic Society for the Study and Protection of the Monk Seal, Athens, Greece; 3 Hellenic Centre for Marine Research, Heraklion, Institute of Oceanography, Crete, 70014, Greece; 4 Department of Biology, School of Sciences and Engineering, Heraklion, University of Crete, Crete, 70013, Greece; 5 Exotic Animal and Wildlife Unit, Clinic of Companion Animals, Faculty of Veterinary Medicine, 11 Voutyra Str, Aristotle University, Thessaloniki, 546-27, Greece; 6 Leibniz Institute for the Analysis of Biodiversity Change, Adenauerallee 127, Museum Koenig Bonn, Bonn, 53113, Germany; 7 Metazoa Phylogenomics and Genome Evolution Lab, Passeig marítim de la Barceloneta 37-49, Institute for Evolutionary Biology (CSIC-UPF), Barcelona, 08003, Spain; 8 National Genomics Infrastructure, Uppsala Genome Center, SciLifeLab, Uppsala, Box 815, 751 08, Sweden; 9 Department of Immunology, Genetics and Pathology, Uppsala University, Uppsala, Box 815, 751 08, Sweden; 10 Department of Cell and Molecular Biology, National Bioinformatics Infrastructure Sweden, Science for Life Laboratory, Uppsala University, Uppsala, 752 37, Sweden; 11 SIB Swiss Institute of Bioinformatics, Amphipôle, Quartier UNIL-Sorge, Lausanne, 1015, Switzerland

**Keywords:** Monachus monachus, genome assembly, European Reference Genome Atlas, Biodiversity Genomics Europe, Earth Biogenome Project, Mediterranean monk seal, Phocidae, Μεσογειακή φώκια

## Abstract

The Mediterranean monk seal, *Monachus monachus*, is the only pinniped that lives in the Mediterranean Sea and one of the rarest marine mammals in the world. The species was recently classified as “vulnerable” by the IUCN, considering an improvement in its overall status. However, the species’ populations have undergone severe bottlenecks due to systematic persecution by humans over the past centuries. Today, the global population of *M. monachus* is estimated to be no more than 1,000 individuals. The Mediterranean monk seal is mainly using marine caves as resting and pupping sites. It is an opportunistic apex predator and as a result its role is considered important for maintaining the structure and function of marine ecosystems. Nowadays, the species is threatened mainly by the destruction of its habitat (due to coastal development, mass tourism, and pollution) and by the depletion of its prey due to overfishing. The Mediterranean monk seal is an emblematic species; its ecological importance and its vulnerable status render its protection and effective management necessary. The entirety of the genome sequence of a female specimen was assembled into 16 contiguous chromosomal pseudomolecules, one sex chromosome (X), and one mitochondrial genome. This chromosome-level assembly encompasses 2.4 Gb, composed of 316 contigs and 275 scaffolds, with contig and scaffold N50 values of 90.9 Mb and 157 Mb, respectively.

## Introduction

**Figure 1.**
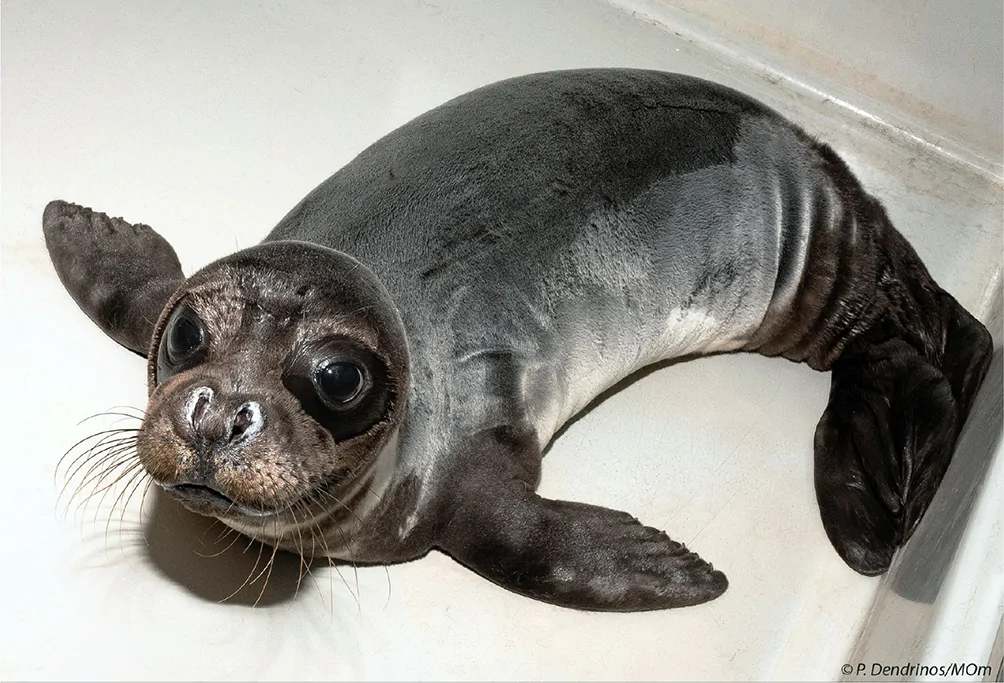
Constantina, the herein sequenced Mediterranean monk seal individual. Photo credit: Panagiotis Dendrinos, MOm/Hellenic Society for the Study and Protection of the Monk Seal.

The Mediterranean monk seal *Monachus monachus*, also known in Greek as Μεσογειακή φώκια, is a monotypic pinniped species that belongs to the family, *Phocidae* (subfamily *Monachinae*) ( Figure 1). It is a medium sized seal with a total body length varying between 2.42 m – 2.51 m and an average weight reaching up to 300 kg ( Karamanlidis et al., 2023; Karamanlidis & Dendrinos, 2023). Recently analysed data support that monk seals probably evolved from the southern hemisphere seals about 12.7 Mya and later moved northward ( Karamanlidis & Dendrinos, 2023; Nebenführ et al., 2025).

The species was once widely distributed in the Mediterranean and Black Seas, and in north Atlantic waters from the Western Sahara and the Canary Islands to Morocco, the Azores and the Madeira archipelago ( Karamanlidis et al., 2023). Today, the distribution of the species is highly fragmented into only three, isolated subpopulations. In the North Atlantic, two subpopulations remain: a larger one at the Cabo Blanco, W. Sahara (approx. 350 individuals) and a much smaller (approx. 30 individuals) at the archipelago of Madeira. In the Mediterranean, the main stronghold of the species, monk seals are found mainly in the eastern part of the basin (approx. 500 individuals), around the islands of the Ionian and the Aegean Seas and the mainland coast of Greece, the western and southern coast of Turkey as well as of Cyprus ( Karamanlidis et al., 2023; Karamanlidis & Dendrinos, 2023; Kurt & Gücü, 2021; Nicolaou et al., 2021). Mediterranean monk seals prefer to use sea caves of a specific morphology to rest and, above all, to give birth and raise their young. This adaptation appears to be the result of the species’ long-term and systematic hunting by humans. The birth of pups in the Mediterranean takes place mainly during the autumn months, peaking in October. Newborns are about 1 m long, weigh 15–26 kg, and nurse for 3–4 months.

The Mediterranean monk seal is the only seal species living in the Mediterranean Sea and one of the rarest marine mammals on the planet. For this reason, it is considered an emblematic species for the conservation of biodiversity in the Mediterranean. It is an opportunistic top predator and therefore its presence is considered of great importance for the functioning and sustainable conservation of marine ecosystems. The protection of the Mediterranean monk seal in its natural environment essentially requires the effective management and protection of marine and coastal ecosystems.

The generation of this reference resource was coordinated by the European Reference Genome Atlas (ERGA) initiative’s Biodiversity Genomics Europe (BGE) project, supporting ERGA’s aims of encouraging transnational cooperation and promoting advances in the application of high-throughput and state-of-the-art genomic technologies to protect and restore biodiversity ( Mazzoni et al., 2023).

## Materials & Methods

ERGA’s sequencing strategy includes Oxford Nanopore Technology (ONT) and/or Pacific Biosciences (PacBio) for long-read sequencing, along with Hi-C sequencing for chromosomal architecture, Illumina Paired-End (PE) for polishing (i.e., recommended for ONT-only assemblies), and RNA sequencing for transcriptomic profiling to facilitate genome assembly and annotation.

### Sample and sampling information

On 10th of October 2024, Tereza Manousaki, Katerina Vasileiadou, Panagiotis Dendrinos, Eleni Tounta and Nikitas Vogiatzis organized the sample blood from one female specimen of *Monachus monachus* in MOm’s Monk Seal Rehabilitation facilities, located at Attica Zoological Park, Spata, Athens, Greece. The species was identified by experts. Sampling was conducted under the permit 104104/3848/06-10-2025 issued by the Hellenic Ministry for Environment and Energy, General Secretariat of Forests. General Directorate of Forests and Forest Environment, Directorate of Forest Management, Department of Wildlife and Hunting Management. The blood sample was taken by an experienced veterinary assistant from the live animal undergoing treatment. The sampling had no impact on the animal’s health status and behaviour. The blood sample was immediately flash-frozen in liquid nitrogen and preserved at −80 °C until DNA extraction.

### Vouchering information

**Figure 2.**
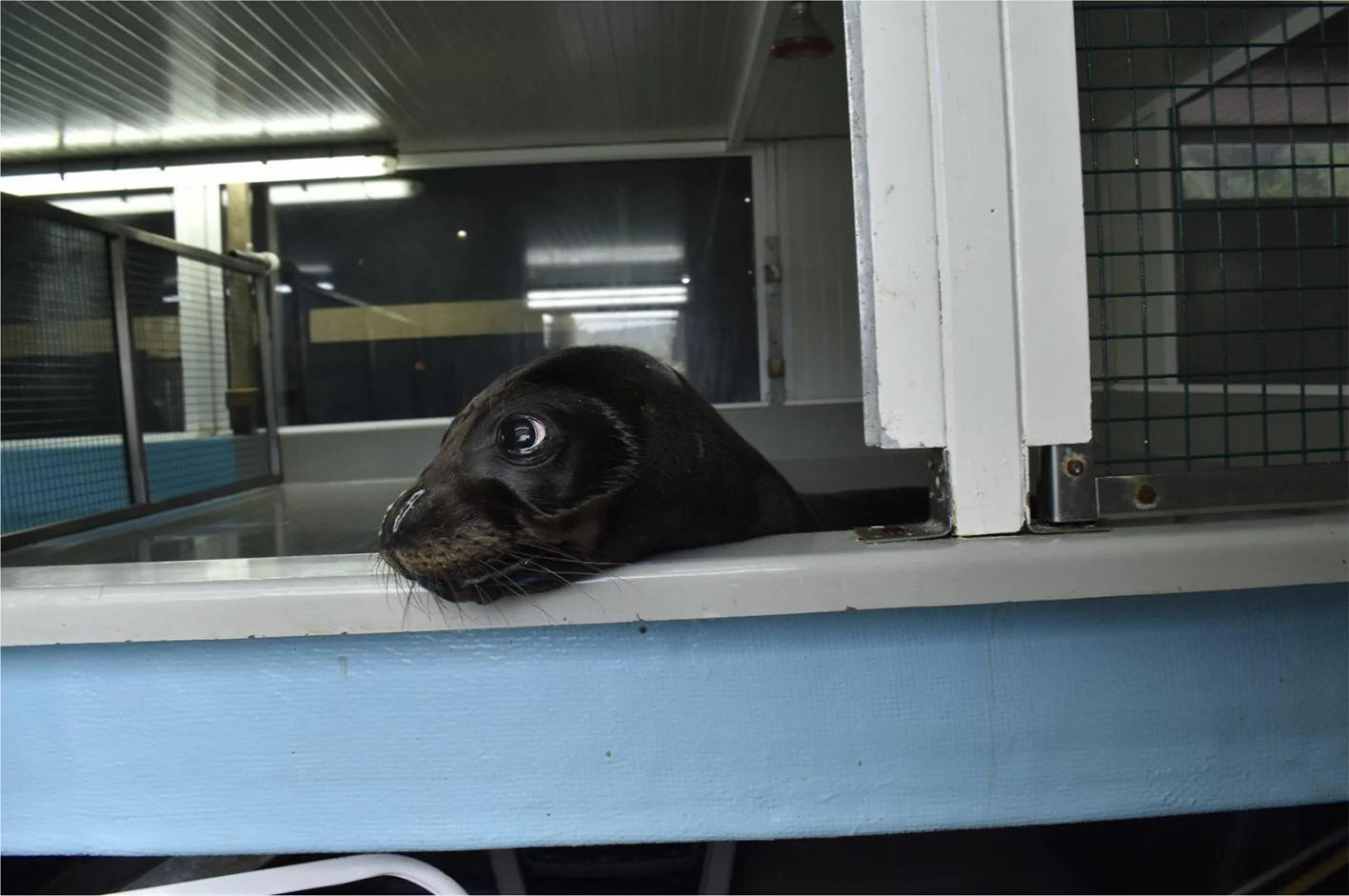
Costantina, BioSample: SAMEA117387578. This picture corresponds to the voucher deposited in BioImage Archive and represents the specimen used here for genome assembly.

Photographs of the specimen have been submitted to BioImage Archive ( https://www.ebi.ac.uk/bioimage-archive/) ( Figure 2) and are here used as a voucher.

### Data availability

*Monachus monachus* and the related genomic study were assigned to Tree of Life ID (ToLID) ‘mMonMoa1’ and all sample, sequence, and assembly information are available under the umbrella BioProject PRJEB96355. The sample information is available at the following BioSample accessions: SAMEA117387579, SAMEA117387581, and SAMEA117387583. The genome assembly is accessible from ENA under accession number GCA_976986665.1 and the annotated genome will be made available through the Ensembl Website ( https://projects.ensembl.org/erga-bge/). Sequencing data produced as part of this project are available from ENA at the following accessions: ERX14538616, ERX16161577, and ERX14869051. Documentation related to the genome assembly and curation can be found in the ERGA Assembly Report (EAR) document available at https://github.com/ERGA-consortium/EARs/tree/main/Assembly_Reports/Monachus_monachus/mMonMoa1. Further details and data about the project are hosted on the ERGA portal at https://portal.erga-biodiversity.eu/data_portal/248254.

### Genetic information

The estimated genome size, based on ancestral taxa, is 2.88 Gb. This corresponds to a diploid genome with an expected haploid number of 17 chromosomes (2n = 34). All information for this species was retrieved from Genomes on a Tree ( Challis et al., 2023).

### DNA/RNA processing

DNA was extracted from 200 μl of frozen blood following PacBio’s instruction described in “Procedure checklist Extracting HMW DNA from human whole blood using Nanobind® kits” (REV04 AUG2024). Quantification was performed using a Qubit dsDNA BR Assay kit (Thermo Fisher Scientific) and integrity was assessed using a FemtoPulse system (Agilent). DNA was stored at 4 °C until usage. RNA was extracted from blood using the TRIzol Reagent and Phasemaker Tubes Complete System (Invitrogen Cat #A33250) following the Invitrogen user guide (Pub. No. MAN0016163 Rev. A.0) except for steps 2.a. and 3.d., which were omitted. RNA was resuspended in 87.5 μl RNase-free water and immediately subjected to DNase treatment followed by purification according to the RNeasy Micro Handbook (pages 74 and 53). Quantity and integrity were assessed using the Agilent 2100 Bioanalyzer instrument. The eluted RNA was stored at −70 °C.

### Library preparation and sequencing

One PacBio SMRTbell library was constructed using the instructions described in “Procedure & Checklist - Preparing whole genome and metagenome libraries using SMRTbell prep kit 3.0” (PN 102–182-700, REV06JAN2025). Prior to library construction, the DNA was sheared using the Megaruptor 3 (Diagenode), followed by a clean-up step using SMRTbell cleanup beads. In total, 3 μg of sheared and cleaned DNA were used for library construction. Size-selection of the ready SMRTbell was performed using the PippinHT system. Primer annealing and polymerase binding were performed using the Revio polymerase kit. Finally, two Revio SMRTcell 25 M were sequenced on the PacBio Revio System. Hi-C was generated from blood as described in the User Guide for Library Preparation for Arima High Coverage HiC Kit (A160186 v02) (Arima Genomics) and sequenced on the NovaSeq X system and XLEAP-SBS sequencing chemistry (Illumina Inc.). Sequencing library was prepared from 100 ng total RNA using the TruSeq stranded mRNA library preparation kit (cat# 20020595, Illumina Inc.) including polyA selection. Unique dual indexes (cat# 20022371, Illumina Inc.) were used. The library preparation was performed according to the manufacturers’ protocol (#1000000040498). The library was sequenced on an Illumina NovaSeq X Plus system with a 25B flow cell using paired-end 150 bp read length and XLEAP-SBS sequencing chemistry. RNA-Seq data are currently being used to generate the genome annotation of *M. monachus.* In total, 91x PacBio data were sequenced to generate the assembly. The Hi-C library failed, and it was not used in scaffolding.

### Genome assembly methods

The genome was assembled using the National Bioinformatics Infrastructure Sweden (NBIS) Earth-Biogenome-Pilot pipeline ( h ttps://github.com/NBISweden/Earth-Biogenome-Project-pilot ), and reference-scaffolded using RagTag v2.1.0 ( Alonge et al., 2022). Briefly, PacBio HiFi reads were assembled using Hifiasm with default parameters ( Cheng et al., 2021). The resulting contigs were screened for contaminants using FCS-GX ( Astashyn et al., 2024) and purged for haplotypic duplications using purge_dups ( Guan et al., 2020). The decontaminated, purged contigs were then reference-scaffolded using RagTag and the genome of the Hawaiian Monk Seal, *Neomonachus schauinslandi*, as reference (GCF_002201575.2). RagTag was run in scaffold mode, and no breaks were introduced in the hifiasm contigs. Prior to reference scaffolding, the contigs of *M. monachus* and the scaffolds of *N. schauinslandi* were analysed for synteny and collinearity using protein-coding genes alignments ( https://github.com/gbdias/quick_synteny). Finally, the mitochondrial genome was assembled as a single circular contig of 16,781 bp using the MitoHiFi pipeline ( Uliano-Silva et al., 2023) and included in the released assembly (GCA_976986665.1).

## Results

### Genome assembly

**Figure 3.**
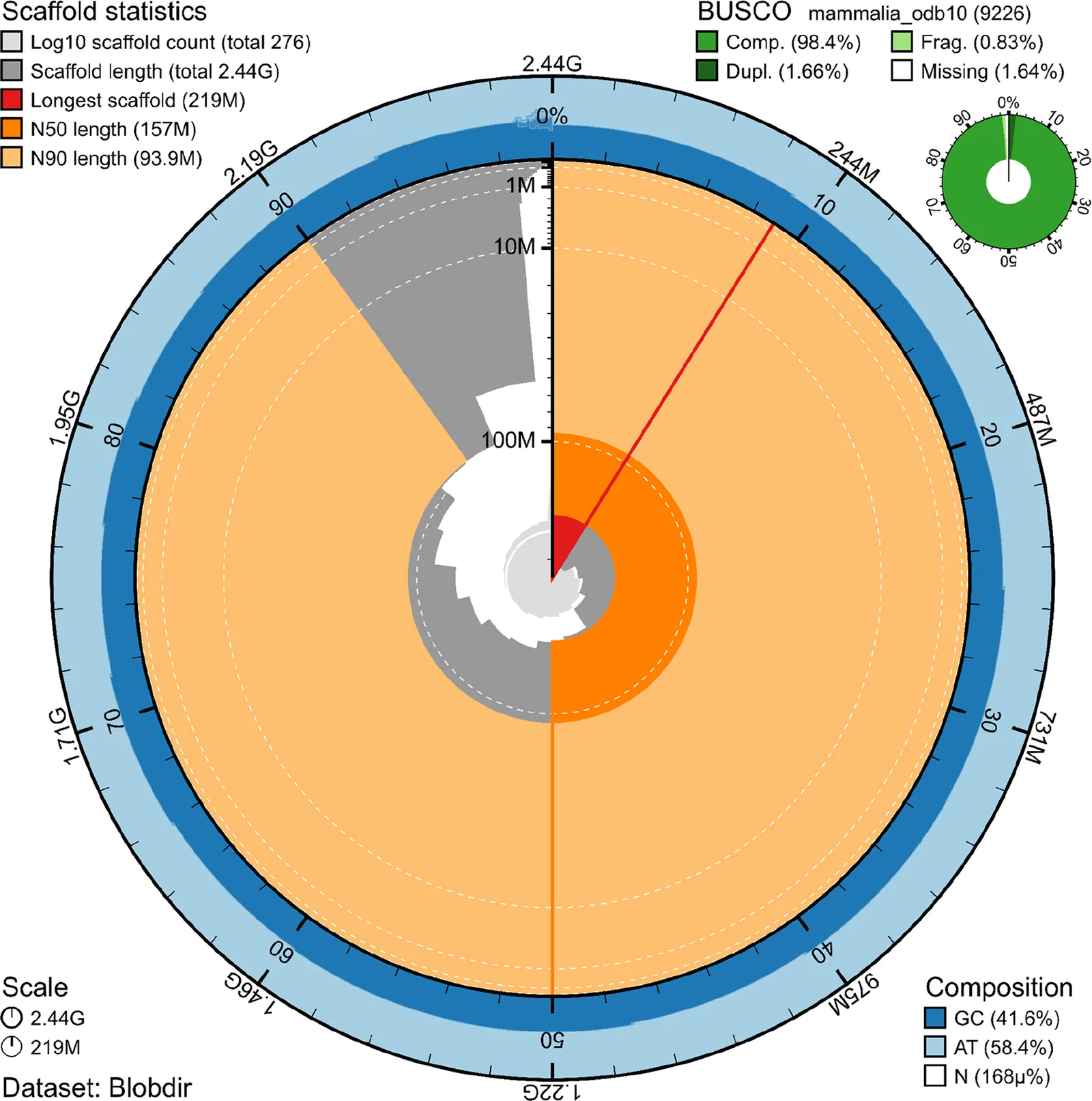
Snail plot summary of assembly statistics. The main plot is divided into 1,000 size-ordered bins around the circumference, with each bin representing 0.1% of the 2,437,322,148 bp assembly including the mitochondrial genome. The distribution of sequence lengths is shown in dark grey, with the plot radius scaled to the longest sequence present in the assembly (219 Mb, shown in red). Orange and pale-orange arcs show the scaffold N50 and N90 sequence lengths (157,001,452 bp and 93,858,021 bp), respectively. The pale grey spiral shows the cumulative sequence count on a log-scale, with white scale lines showing successive orders of magnitude. The blue and pale-blue area around the outside of the plot shows the distribution of GC, AT, and N percentages in the same bins as the inner plot. A summary of complete, fragmented, duplicated, and missing BUSCO genes found in the assembled genome from the Mammalia database (odb10) is shown in the top right.

The genome assembly has a total length of 2,437,322,148 bp in 276 scaffolds including the mitogenome ( Figure 3), with a GC content of 41.5%. The assembly has a contig N50 of 90,875,510 bp and L50 of 10 and a scaffold N50 of 157,001,452 bp and L50 of 7. The assembly has a total of 41 gaps, totaling 4.1 kb in cumulative size. The single-copy gene content analysis using the Mammalia database (odb10) with BUSCO v5.8.0 ( Manni et al., 2021) resulted in 98.4% completeness (96.7% single and 1.7% duplicated). 99.47% of reads *k- *mers were present in the assembly and the assembly has a base accuracy Quality Value (QV) of 68.6 as calculated by Merqury ( Rhie et al., 2020).

## Author contributions

TM, KV and PD coordinated the project; PD, ET, NV, AK collected the species; PD, ET, NV, AK identified the species; TM, PD, KV, XS, CST, TD, ET, NV, AK sampled and preserved biological material and provided metadata; AsB, RM, RF, and NE provided support in sampling, shipping of biological material, metadata collection, and management; M-BM extracted DNA and RNA under supervision of OVP; CTR and IB performed raw data processing and data QC; GBD performed genome assembly and curation; CB generated the analysis and report. All authors contributed to the writing, review, and editing of this genome note and read and approved the final version.

## References

[ref1] Alonge M Lebeigle L Kirsche M : Automated assembly scaffolding using RagTag elevates a new tomato system for high-throughput genome editing. *Genome Biol.* 2022;23(1):258. 10.1186/s13059-022-02823-7 36522651 PMC9753292

[ref2] Astashyn A Tvedte ES Sweeney D : Rapid and sensitive detection of genome contamination at scale with FCS-GX. *Genome Biol.* 2024;25(1):60. 10.1186/s13059-024-03198-7 38409096 PMC10898089

[ref3] Challis R Kumar S Sotero-Caio C : Genomes on a Tree (GoaT): A versatile, scalable search engine for genomic and sequencing project metadata across the eukaryotic tree of life. *Wellcome Open Research.* 2023;8:24. 10.12688/wellcomeopenres.18658.1 36864925 PMC9971660

[ref4] Cheng H Concepcion GT Feng X : Haplotype-resolved de novo assembly using phased assembly graphs with hifiasm. *Nat. Methods.* 2021;18(2):170–175. 10.1038/s41592-020-01056-5 33526886 PMC7961889

[ref5] Guan D McCarthy SA Wood J : Identifying and removing haplotypic duplication in primary genome assemblies. *Bioinformatics.* 2020;36(9):2896–2898. 10.1093/bioinformatics/btaa025 31971576 PMC7203741

[ref6] Karamanlidis AA Dendrinos P : Mediterranean Monk Seal Monachus monachus (Hermann, 1779). Hackländer K Zachos FE , editors. *Handbook of the Mammals of Europe.* Springer International Publishing;2023; pp.1–21. 10.1007/978-3-319-65038-8_143-1

[ref7] Karamanlidis AA Dendrinos P Fernandez de Larrinoa P : Monachus monachus. The IUCN Red List of Threatened Species 2023; e. T13653A238637039. 2023. Reference Source

[ref8] Kurt M Gücü AC : Demography and population structure of Northeastern Mediterranean monk seal population. *Mediterr. Mar. Sci.* 2021;22(1):79–87.

[ref9] Manni M Berkeley MR Seppey M : BUSCO update: Novel and streamlined workflows along with broader and deeper phylogenetic coverage for scoring of eukaryotic, prokaryotic, and viral genomes. *Mol. Biol. Evol.* 2021;38(10):4647–4654. 10.1093/molbev/msab199 34320186 PMC8476166

[ref10] Mazzoni CJ Ciofi C Waterhouse RM : Biodiversity: An atlas of European reference genomes. *Nature.* 2023;619(7969):252–252. 10.1038/d41586-023-02229-w 37433931

[ref11] Nebenführ M Hamadou AB Aguilar A : Mediterranean monk seal (Monachus monachus) and leopard seal (Hydrurga leptonyx) de novo genomes to study the demographic history and genetic diversity of southern seals. *BMC Biol.* 2025;23(1):102. 10.1186/s12915-025-02207-w 40241156 PMC12004778

[ref12] Nicolaou H Dendrinos P Marcou M : Re-establishment of the Mediterranean monk seal Monachus monachus in Cyprus: Priorities for conservation. *Oryx.* 2021;55(4):526–528.

[ref13] Rhie A Walenz BP Koren S : Merqury: Reference-free quality, completeness, and phasing assessment for genome assemblies. *Genome Biol.* 2020;21(1):245. 10.1186/s13059-020-02134-9 32928274 PMC7488777

[ref14] Uliano-Silva M Ferreira JGRN Krasheninnikova K : MitoHiFi: A python pipeline for mitochondrial genome assembly from PacBio high fidelity reads. *BMC Bioinformatics.* 2023;24(1):288. 10.1186/s12859-023-05385-y 37464285 PMC10354987

