# Supplementary material for: ERGA-BGE reference genome of the Mediterranean monk seal ( Monachus monachus), an IUCN Vulnerable species
Source: Open Res Eur. 2026 Jun 9;6:195. [Version 1] doi: 10.12688/openreseurope.23936.1 (PMC13560844; doi:10.12688/openreseurope.23936.1)
Supplement: Peer review history

# Reviewer report: Reviewer response for version 1

**DOI:** 10.21956/openreseurope.25914.r75901

**Published:** 2026-08-07

**Authors:** Anna Brüniche-Olsen

**Affiliations:** 1 University of Copenhagen, Copenhagen, Denmark

The manuscript provides a high quality reference genome for the monk seal, a species of conservation concern, where this genomic resource will contribute to future genomic studies of the focal and other marine mammal species.  Overall the manuscript is well written and concise. I only have a few comments, that I hope the authors will consider.

 INTRODUCTION

 A map with past and current ranges would help the reader. In the second paragraph is detailed information on past distribution and current population structure. It would be good to have a visual for this, as it would also provide context for the substantial population decline and conservation listing.

 Its described that the monk seal uses sea caves to rest, give birth and raise young in. Are these caves locally protected, so the species can raise young undisturbed by humans?

 What is an “opportunistic top predator”? Do you mean, that when no other large predators are around, then the monk seal moves up in the trophic cascade? I would label it as either apex or meso predator, depending on that the majority of the species are.

 MATERIALS AND METHODS

 DNA/RNA processing: delete “frozen”. (DNA was extracted from 200ul of **frozen **blood…”).

 Library prep and seq: In the last sentence is stated that the Hi-C library failed. What implications does this have for the quality of the final genome assembly? Would it be beneficial to construct a new Hi-C library and use it for scaffolding?

Are sufficient details of methods and materials provided to allow replication by others?

Yes

Is the rationale for creating the dataset(s) clearly described?

Yes

Are the datasets clearly presented in a useable and accessible format?

Yes

Are the protocols appropriate and is the work technically sound?

Yes

Reviewer Expertise:

Population genomics, conservation genomics, macrogenetics

I confirm that I have read this submission and believe that I have an appropriate level of expertise to confirm that it is of an acceptable scientific standard.

# Reviewer report: Reviewer response for version 1

**DOI:** 10.21956/openreseurope.25914.r75755

**Published:** 2026-07-17

**Authors:** Luis A Escobedo-Morales

**Affiliations:** 1 Universidad Nacional Autónoma de México, Mexico City, Mexico

The manuscript provides a valuable genomic resource for future studies on a relevant marine mammal species. The manuscript is well-rounded, and the Methods are clearly described. Some numbers must be revised to ensure consistency between Figure 3 and the manuscript text. An additional review of the language would be helpful to round out scores and avoid wordy phrases. The document only needs a review of some details, but it has sufficient quality and merit for the immediate publication of the report. It would be interesting to briefly discuss the validation tools, including the failed Hi-C library.

 I list a few suggestions to enhance readability for the readers:

 Introduction

 First paragraph

 Line 2: Please remove the comma after "family". Also include Order for better reference for readers of the species taxonomy.

 Line 2: Please change "medium sized" to "medium-sized".

 Second paragraph

 Line 3: Please change "into only three, isolated subpopulations" to "with only three isolated subpopulations".

 Lines 4, 5, and 6: Please substitute "approx." for "approximately".

 Line 7: Please change "as well as of" to "and".

 Line 9: Please change "sea caves of" to "sea caves with".

 Lines 10 and 11: Please change "The birth of pups in the Mediterranean takes place" to "In the Mediterranean, pupping occurs".

 Third paragraph

 Line 2: Please change "the conservation of biodiversity" to "biodiversity conservation".

 Line 4: Please change "The protection of" to "Protecting".

 Materials & Methods

 Second paragraph (Sample and sampling information)

 Line 1: Please change "On 10th of October 2024" to "On October 10, 2024".

 Line 2: Please change "organized the sample blood" to "collected a blood sample".

 Line 2: Please change "one female specimen of Monachus monachus" to "a female Monachus monachus specimen".

 Line 3: Please remove "located".

 Third paragraph (Vouchering information)

 Line 2: Please change "here used" to "used here".

 Fourth paragraph (Data availability)

 Line 2: Please change "The sample" to "Sample".

 Line 8: Please remove "document".

 Fifth paragraph (Genetic information)

 Line 1: Please clarify this genome size estimate (2.88 Gb) and standardize the decimal formatting throughout the manuscript (2.44 Gb).

 Sixth paragraph (DNA/RNA processing)

 Line 7: Please change "subjected to DNase treatment followed by purification" to "treated with DNase, then purified"

 Seventh paragraph (Library preparation and sequencing)

 Line 1: Please change "using the instructions described in" to "following the instructions in".

 Line 3: Please change "the DNA" to "DNA".

 Line 3: Please change "followed by a clean-up step using" to "and then cleaned with".

 Line 9: Please change "Sequencing" to "The sequencing".

 Eighth paragraph (Genome assembly methods)

 Line 3: Please remove "Briefly,".

 Line 5: Please change "purge_dups" to "the purge_dups tool".

 Line 8: Please change "analysed" to "analyzed".

 Figure 3. Please review the scaffold count (276 in the figure, 275 in the Abstract).

 Results

 Line 1: Please verify that the scaffold count is correct in the Abstract, Methods, and Results sections. Also, verify that the numbers in Figure 3 and the manuscript match.

 Line 3: Please change "totaling 4.1 kb in cumulative size" to "for a cumulative size of 4.1 kb".

Are sufficient details of methods and materials provided to allow replication by others?

Yes

Is the rationale for creating the dataset(s) clearly described?

Yes

Are the datasets clearly presented in a useable and accessible format?

Yes

Are the protocols appropriate and is the work technically sound?

Yes

Reviewer Expertise:

Phylogenomics, museomics, high-throughput sequencing.

I confirm that I have read this submission and believe that I have an appropriate level of expertise to confirm that it is of an acceptable scientific standard.
